# Targeting MDK alleviates bone loss via dual regulation of osteogenic differentiation and inflammatory cytokine expression

**DOI:** 10.1016/j.gendis.2025.101931

**Published:** 2025-11-10

**Authors:** Xieyidai Ruze, Yutong Hu, Xiongyi Wang, Houfu Lai, Ruizhi Zhang, Sheng Pan, Jiajun Zhang, Yike Wang, Simin Yun, Ying Xu, Junjie Li, Youjia Xu

**Affiliations:** a Department of Orthopedics, Department of Osteoporosis, The Second Affiliated Hospital of Soochow University, Suzhou, Jiangsu 215004, China; b The First Affiliated Hospital of Zhejiang Chinese Medical University (Zhejiang Provincial Hospital of Chinese Medicine), Hangzhou, Zhejiang 310006, China; c Jiangsu Key Laboratory of Neuropsychiatric Diseases and Cambridge-Su Genomic Resource Center, Medical College of Soochow University, Suzhou, Jiangsu 215000, China

**Keywords:** Growth factors, Inflammatorycytokines, MDK, Osteoblast, Targeted therapy

## Abstract

Growth factors are bioactive molecules that play crucial roles in regulating growth, development, and disease processes, both locally and systemically. Identifying growth factors involved in bone homeostasis and targeting them is a key strategy for treating bone metabolic diseases. In this study, we observed significantly elevated serum levels of midkine (MDK) in patients with postmenopausal osteoporosis and in ovariectomized mice, based on clinical data and animal experiments. We also identified a negative correlation between MDK levels and bone mineral density. The small molecule inhibitor of MDK, iMDK, effectively mitigated estrogen deficiency-induced bone loss by promoting bone formation and inhibiting inflammatory factors. Our *in vitro* experiments further revealed that recombinant MDK protein dose-dependently inhibited osteogenic differentiation. Transcriptome analysis showed that recombinant MDK protein affected osteogenic differentiation through the PI3K/AKT signaling pathway. Additionally, it increased the expression of inflammatory cytokines, including IL-6, TNF-α, and IL-1β, via the NF-κB signaling pathway. These findings suggest that MDK could serve as a novel therapeutic target for postmenopausal osteoporosis, and that iMDK may be a promising therapeutic candidate.

## Introduction

Osteoporosis, a common bone metabolic disorder, is frequently observed in older adults, postmenopausal women, and individuals with chronic inflammatory conditions.^1^^,^^2^ This condition is characterized by reduced bone mass and deterioration of bone microarchitecture, which increases the risk of pathological fractures.^3^ The balance between osteoblast-driven bone formation and osteoclast-mediated bone resorption is crucial in the progression of osteoporosis. Disruption of this balance leads to the development of the disease. Various factors, including insufficient estrogen levels, inflammation, cellular aging, oxidative stress, and epigenetics, influence osteoblast activity.^4^ Additionally, growth factors and secretory proteins within the body can affect bone remodeling, contributing to osteoporosis. For example, the interaction between receptor activator of nuclear factor kappa-B (RANK) and its ligand RANKL promotes osteoclast differentiation and activity. Denosumab, which inhibits the RANKL-RANK interaction, effectively reduces osteoclast development and resorption but may present risks, such as infection and hypocalcemia.^5^ Similarly, sclerostin (SOST) negatively regulates osteoblast activity through the Wnt/β-catenin pathway. This regulation can be counteracted by romosozumab to enhance bone formation, though this treatment has potential limitations, including restricted duration and cardiovascular concerns.^6^ Therefore, a comprehensive understanding of how growth factors and secretory proteins influence osteoporosis, along with the mechanisms of inhibitors and targeted therapies, is crucial for effective treatment.

Inflammatory cytokines play a key role in the pathophysiology of osteoporosis.^4^^,^^7^ These cytokines exert bidirectional effects on bone homeostasis. Specifically, cytokines such as interleukin-6 (IL-6), interleukin-1β (IL-1β), and tumor necrosis factor-alpha (TNF-α) primarily promote bone resorption, contributing to the development of osteoporosis.^8^^,^^9^ At the same time, these cytokines can suppress osteoblast activity, thereby hindering bone formation. Research shows that chronic inflammatory conditions, often associated with aging and estrogen deficiency, lead to increased cytokine release. This release inhibits osteoblast differentiation and bone formation while stimulating osteoclast proliferation, thereby worsening the progression of osteoporosis.^10^ Chronic inflammatory disorders like rheumatoid arthritis and systemic lupus erythematosus exemplify conditions that heighten the risk of osteoporosis *in vivo*. These disorders affect systemic bone metabolism through inflammatory cytokines that alter local bone tissue and blood circulation. Mechanistically, these cytokines reduce bone formation signals such as Wnt/β-catenin while promoting pathways like nuclear factor-kappa B (NF-κB) and phosphoinositide 3-kinase (PI3K), which inhibit osteoblast differentiation. Therefore, it is crucial to maintain a balance between bone production and resorption and to regulate the suppression of osteoblasts by inflammatory cytokines.

The growth factor midkine (MDK) belongs to the family of heparin-binding growth factors and plays a role in both disease onset and the maintenance of healthy tissue function. MDK is expressed in various tumor cells, where it promotes tumor cell growth and survival.^11^^,^^12^ Li et al demonstrated that MDK enhances tumor cell proliferation, angiogenesis, and epithelial–mesenchymal transition in breast cancer bone metastasis through its interaction with stromal cells (including FAP^+^ inflammatory cells and myofibroblasts), thereby promoting metastatic potential. Elevated MDK expression shows significant correlation with tumor metastasis and poor patient survival prognosis.^13^ A small molecule inhibitor of MDK, iMDK, has shown therapeutic effects on tumor cells.^14^, ^15^, ^16^, ^17^, ^18^ Studies on MDK's role in bone metabolism have demonstrated that animals lacking MDK exhibit increased trabecular bone growth.^19^ In mice, the absence of MDK enhances the cortical bone's synthetic metabolic response to mechanical strain.^20^ Additionally, patients with knee osteoarthritis have significantly higher levels of MDK in their serum and synovial fluid compared with healthy controls.^21^ Research on the effects of MDK protein on osteoblasts has primarily focused on the Wnt/β-catenin signaling pathway, while the role of iMDK in bone metabolism remains unexplored. Previous studies have shown that MDK activates the NF-κB and PI3K/protein kinase B (AKT) signaling pathways in tumor cells, promoting cell growth.^22^, ^23^, ^24^ Furthermore, MDK has been shown to facilitate the migration of inflammatory leukocytes in rheumatoid arthritis^25^ and to assist in the recruitment of neutrophils and macrophages to inflammatory sites during acute inflammation.^26^^,^^27^ However, the regulation of inflammatory cytokines by MDK in relation to bone formation and its activation of the PI3K/AKT and NF-κB signaling pathways have been less studied. Moreover, the potential of targeting MDK for the treatment of osteoporosis induced by estrogen deficiency has not been explored.

In summary, this study established a correlation between serum MDK protein levels and osteoporosis using clinical data and animal models. Based on this finding, we assessed the anti-osteoporotic effects of the MDK inhibitor iMDK and explored MDK's role in bone formation and its regulation of inflammatory cytokines through cellular experiments. Additionally, we validated the specific mechanisms involved using transcriptome sequencing and further cellular assays. Our research offers insights into the distinct mechanisms by which MDK inhibits osteogenic growth and highlights the potential of targeting MDK inhibition as a therapeutic strategy for treating osteoporosis.

## Materials and methods

### Chemicals and reagents

We dissolved MDK (HY–P700138AF, MCE, USA) in phosphate-buffered saline solution (PBS) to a concentration of 100 ng/mL and stored it at −20 °C. We obtained penicillin-streptomycin solution (Cat#15070063), alpha minimum essential medium (α-MEM) cell culture media (Cat#12571063), and fetal bovine serum (Cat#A5669701) from Thermo Fisher Scientific (Wuhan, China). β-Glycerophosphate disodium salt hydrate (Cat#M3837) was purchased from AbMole (USA). Ascorbic acid (Cat#50-81-7) came from Sigma Aldrich (USA). LY294002 (Cat#154447-36-6) and BAY11-7082 (Cat#19542-67-7) were obtained from TargetMol (USA). We purchased the Mouse P1NP (Procollagen 1 N-Terminal Propeptide) ELISA Kit (Cat#e-el-m0233), Mouse IL-6 ELISA Kit (Cat#e-el-m0044), Mouse TNF-α ELISA Kit (Cat#e-el-m3063), and Mouse IL-1β ELISA Kit (Cat#e-el-m0037) from Elabscience (Wuhan, China).

### Serum sample collection from patients and the enzyme-linked immunosorbent test (ELISA)

We obtained serum samples from postmenopausal female patients between September 2022 and March 2023 at the Second Affiliated Hospital of Soochow University. Inclusion criteria for the study were: age ≥50 years; postmenopausal status; complete medical history; and completion of both serum collection and bone mineral density (BMD) testing. We excluded patients who were under 50 years of age, had incomplete medical histories or test indicators, were diagnosed with hyperthyroidism, hyperparathyroidism, Cushing's syndrome, renal dysfunction, or malnutrition, or had used bone-metabolizing drugs within the previous six months (*e.g.*, glucocorticoids, aromatase inhibitors, thyroid hormones, diuretics, or immunosuppressants). Patients were categorized into two groups based on their dual-energy X-ray absorptiometry T-scores: the osteoporosis group (T-score < −2.5 at any measurement site, either lumbar spine or hip) and the normal bone density group (T-score ≥ −1 at both measurement sites). Each group consisted of 42 patients, with no significant differences in age or body mass index between the groups. The Ethics Committee approved the study on August 29, 2022 (approval number: JD-LK-2022-096-02). We conducted ELISA experiments using Elabscience kits according to the manufacturer's protocol.

### Animals

The Soochow University Animal Welfare and Ethics Committee approved all protocols for animal experiments (No. 202311A0593) before the use of animals. We used 8-week-old female C57BL/6J mice (18–22 g) as experimental subjects. These mice were obtained from Changzhou Cavens Laboratory Animal Co., Ltd., China, and housed in Soochow University's specific pathogen-free animal experiment center. We sterilized the bedding and feed by irradiation and kept the mice in individually ventilated cages.

### Ovariectomy (OVX) model mice

We established three groups of female C57BL/6J mice: OVX plus vehicle (OVX plus PBS), iMDK-treated OVX (OVX plus iMDK), and sham-operated (sham). Following our previous method,^28^ we performed ovariectomy on the OVX groups. One week later, the OVX plus iMDK group received intraperitoneal injections of iMDK (9 mg/kg, 99.77% purity, TargetMol) three times a week for eight weeks (Mondays, Wednesdays, and Fridays at 10 a.m.). We dissolved iMDK in dimethyl sulfoxide (41.4 mg/kg stock) and diluted it in PBS before administration. At the end of the treatment, we collected serum samples and bilateral femurs and tibias for analysis.

### OVX mouse serum ELISA assay

Eight weeks after performing ovariectomy, the mouse serum was collected by orbital blood sampling. Whole blood was allowed to stand for 30 min at room temperature and then centrifuged at 3000 rpm at 4 °C for 15 min to obtain the supernatant, which was stored in portions at −80 °C for reserve. An ELISA kit was purchased from Jonlnbio (Cat# JL25352-96T; Shanghai, China). The sandwich ELISA begins with 90 min incubation of 100 μL standards/samples at 37 °C in pre-coated plates. After aspiration, 100 μL biotinylated detection antibody (1:100 dilution of 100 × concentrate) was added to continue incubation at 37 °C for 60 min. After 3 washes with 350 μL wash buffer (25 × concentrate diluted in 720 mL distilled water), the sample was subjected to incubation with 100 μL horseradish peroxidase conjugate (1:100 dilution) at 37 °C for 30 min. Following 5 washes, 90 μL 3,3′,5,5′-tetramethylbenzidine (TMB) substrate was added to continue incubation in the dark at 37 °C for 15 min. The incubation was terminated with 50 μL stop solution, and reading at 450 nm was immediately performed.

### Immunofluorescence staining

Eight weeks after performing ovariectomy, bone tissue for immunofluorescence experiments was obtained from the mouse femoral stem. Paraffin sections were dewaxed and rehydrated by gradient ethanol and subjected to antigen repair (primary antibody: MDK, 11009-1-AP, dilution ratio: 1:300; secondary antibody: CY3-labelled goat anti-rabbit IgG), with controlled evaporation of buffer during the process. After natural cooling, the sections were washed 3 times with PBS (pH 7.4) on a decolorizing shaker. Tissue sections were slightly shaken dry and then circled with a histochemical pen and closed with a drop of bovine serum albumin for 30 min. The primary antibody was incubated in a wet box at 4 °C overnight, and the corresponding secondary antibody was washed with PBS and incubated at room temperature in the dark for 50 min. The nuclei of the cells were re-stained with 4′,6-diamidino-2-phenylindole (DAPI) for 10 min, and finally, the sections were sealed with anti-fluorescence quenching sealer, and the images were captured according to the set parameters.

### Analysis of micro-computed tomography (Micro-CT)

Eight weeks after performing ovariectomy, we fixed femora in 4% paraformaldehyde at room temperature for 24 h, then examined them *ex vivo* using a micro-CT scanner (Skyscan1174v2, Bruker). We analyzed the region below the growth plate of the distal femur. The scanner operated with a resolution of 10.3 μm, a voltage of 50 kV, a current of 800 mA, and a rotation step of 0.5°. We analyzed several bone morphometric characteristics, including BMD (g/cm^3^), trabecular thickness (Tb.Th), bone volume fraction (BV/TV), trabecular number (Tb.N), and bone surface area (BS).^29^

### Analysis and staining of histology

We decalcified the separated tibiae and femora in ethylenediaminetetraacetic acid (EDTA) on a rotating shaker for four weeks. After decalcification, we dried the samples, embedded them in paraffin, and sectioned them into 5–7 μm slices. We then stored the sections in 4% paraformaldehyde for a full day. Subsequently, we performed MDK immunofluorescence staining, osteocalcin (OCN) immunohistochemistry staining, and hematoxylin-eosin staining on the sections. We scanned the stained tissues using a Panoramic MIDI digital slide scanner and processed the images with CaseViewer software.

### Cultivation and differentiation of cells

Procell Life Science & Technology Co., Ltd. (Wuhan, China) supplied the pre-osteoblastic cell line MC3T3-E1 Subclone 14. We cultured the cells at 37 °C with 5% CO_2_ in α-MEM, which was supplemented with 10% fetal bovine serum and 1% penicillin-streptomycin. We passaged the cells until they reached 90% confluence. To promote osteogenic differentiation, we added 50 μg/mL ascorbic acid, 10 mmol/L β-glycerophosphate, and 10 nmol/L dexamethasone to α-MEM and maintained this condition for seven days.

### Assay for cell viability

We used the Cell Counting Kit-8 (CCK-8, Elabscience) to assess cell viability. We seeded 1 × 10^4^ MC3T3-E1 cells per well in a 96-well plate and incubated them for 24 h. After adding various concentrations of MDK (0, 100, 300, 600, and 900 ng/mL), we continued to incubate the cells at 37 °C with 5% O_2_ for 48 h. The absorbance was measured at 450 nm using a microplate reader to determine cell viability.

### Protein extraction, quantification, and western blotting

For cellular protein extraction, we seeded 5 × 10^6^ MC3T3-E1 cells per well in 6-well plates. Cells were treated with recombinant MDK at the specified concentrations while undergoing osteogenic differentiation. After 7 days, proteins were extracted, and their concentrations were measured using the Biosharp BCA assay kit. Western blotting was performed, with internal references used for normalization. Protein band intensities were quantified using ImageJ software.

For mouse bone tissue total protein extraction, the bilateral femurs of 8-week-old C57BL/6 mice were immediately isolated, and the bone marrow cavity was flushed with pre-chilled PBS until the bone appeared white. The marrow-depleted femurs were transferred to a pre-cooled mortar and immediately covered with liquid nitrogen. When the liquid nitrogen began to vaporize, the bones were rapidly ground using a pestle, with continuous supplementation of liquid nitrogen to maintain a low temperature, until the bone tissue was pulverized into a fine powder. The bone powder was then transferred to a pre-chilled 1.5 mL microcentrifuge tube, and RIPA lysis buffer was added at a tissue-to-lysis buffer ratio of 1:10. The mixture was centrifuged at 12,000 *g* at 4 °C for 15 min. The supernatant was carefully collected into a new microcentrifuge tube, and protein concentration was determined using the BCA assay, followed by Western blotting analysis. The primary antibody was diluted to 1:1000.

### Real-time quantitative PCR with RNA extraction

After 7 days of osteogenic differentiation with MDK treatment, total RNA was extracted from MC3T3-E1 cells using TRIzol reagent, followed by chloroform phase separation. RNA quantity and quality were assessed by NanoDrop-2000 spectrophotometer (A260/280 ratio >1.8). cDNA was synthesized using Novoprotein cDNA Synthesis Super Mix. Quantitative PCR was performed with Novoprotein SYBR Green reagent on a Viia7 instrument using primers listed in Table S1. Gene expression levels were calculated by the 2^–ΔΔCT^ method with β-actin as the internal control.

### Alkaline phosphatase staining (ALP) and alizarin red S staining (ARS)

We seeded MC3T3-E1 cells at predetermined densities in osteogenic induction media and cultivated them for either 14 or 21 days. We conducted ALP staining with a Beyotime kit (C3206) and ARS staining according to the manufacturer's instructions using ARS solution (ALIR-10001, Cyagen). We observed mineralized nodules with an Olympus IX73 inverted microscope and quantitatively analyzed the optical density of the nodules using ImageJ software.

### ALP activity assay

We seeded MC3T3-E1 cells at a predetermined density, treated them with MDK, and allowed them to develop for 14 days in osteogenic induction medium. We measured ALP activity according to the manufacturer's instructions using a kit (P0321S, Beyotime, Shanghai, China). We recorded absorbance at 405 nm and adjusted the final ALP activity based on protein concentration.

### RNA sequencing and bioinformatics analysis

Prepared cells were lysed for RNA extraction and quality control. Transcriptome sequencing was performed using the BGI DNBSEQ-T7 platform. Total RNA was extracted and quality-checked (RIN ≥8.0) following BGI's standard protocols, and strand-specific libraries were constructed using the BGISEQ-500 kit (insert size: 300 bp). Sequencing was conducted with the combinatorial probe-anchor synthesis (cPAS) technology (PE150, ≥40 M reads/sample). Raw data were filtered using SOAPnuke, aligned to the GRCh38 reference genome with HISAT2, and quantified using RSEM. Differential expression analysis was performed with DESeq2 (|log2fold-change| > 1, false discovery rate <0.05), and functional enrichment was analyzed via the Dr. Tom platform.

### Detection of intracellular reactive oxygen species levels

After treating the cells with 600 ng/mL of recombinant MDK protein for 2 h, the cells were incubated with DCFH-DA(S0033S, Beyotime, Shanghai, China) for 25 min to generate fluorescent DCF. The DCF fluorescence was then observed using a fluorescence microscope under excitation at 488 nm and emission at 525 nm.

### Statistical analysis

We conducted experiments with three or more replicates. We present the results as mean ± standard error of the mean. We performed statistical analysis using GraphPad Prism 9. To determine significance, we used either one-way ANOVA or an unpaired *t*-test. *p*-values <0.05 were considered statistically significant.

## Results

### Identifying a negative correlation between MDK levels and BMD

**Figure 1:**
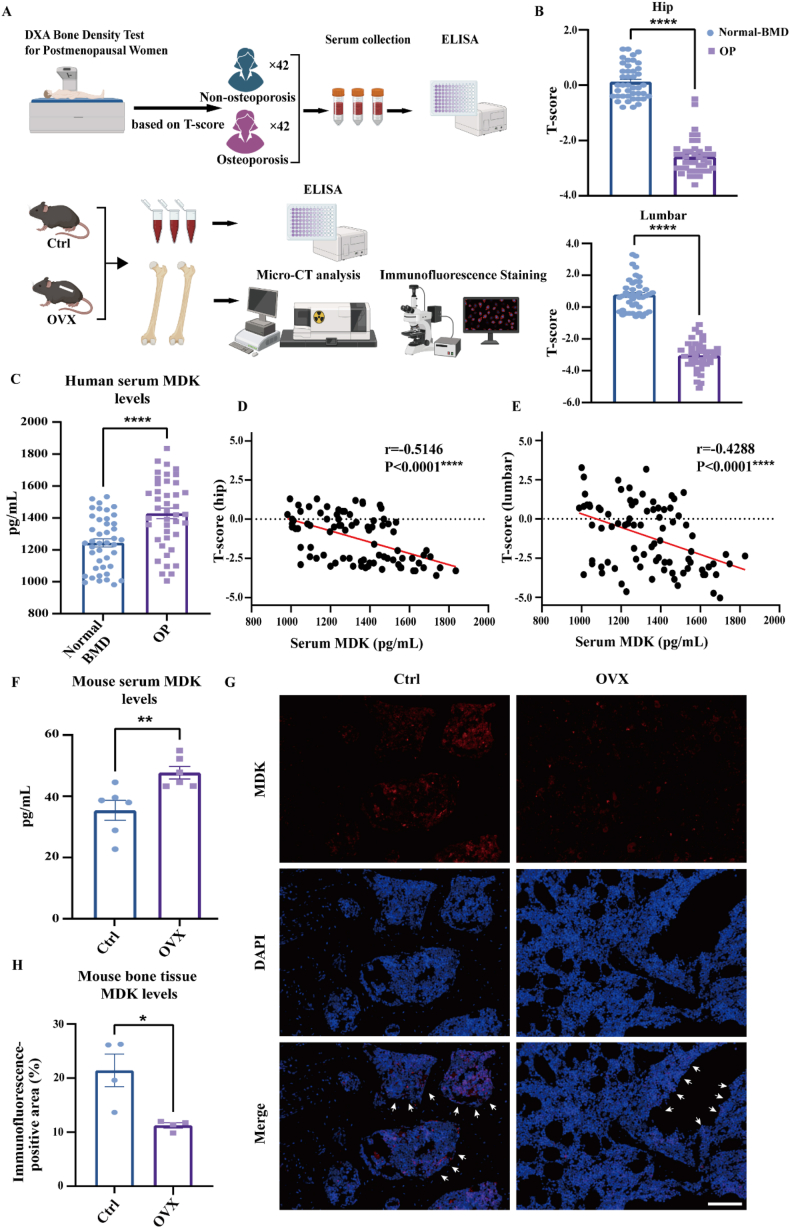
There is a negative correlation between serum MDK levels and BMD. **(A)** Schematic of the experimental workflow for detecting MDK expression in postmenopausal osteoporosis and in ovariectomized mice. **(B)** Statistical analysis of T-score differences between hip and lumbar spine BMD in patients (Normal-BMD *vs*. OP groups). Inter-group comparisons were analyzed by the Wilcoxon signed-rank test. **(C)** Serum MDK protein concentrations were measured using ELISA. Inter-group comparisons were analyzed by a two-tailed unpaired Student's *t*-test (for normally distributed data with equal variance). Normal BMD: normal bone mineral density group (*n* = 42); OP, osteoporosis group (*n* = 42). **(D, E)** Pearson correlation analysis of the relationship between serum MDK concentration and hip BMD T-score (D) and lumbar BMD T-score (E). **(F)** Serum MDK protein concentrations in control and OVX mice were measured using ELISA. Inter-group comparisons were analyzed by a two-tailed unpaired Student's *t*-test (for normally distributed data with equal variance). **(G, H)** Detection and quantitative analysis of MDK protein expression in bone sections of control and OVX mice using immunofluorescence. Inter-group comparisons were analyzed by a two-tailed unpaired Student's *t*-test (for normally distributed data with equal variance). Scale bar, 100 μm ∗*p* < 0.05, ∗∗*p* < 0.01, and ∗∗∗∗*p* < 0.0001.

To explore the correlation between MDK levels and BMD, we collected serum from postmenopausal osteoporosis patients and established an ovariectomized mouse model to assess MDK expression in serum and bone tissues (Fig. 1A). We first collected serum and BMD measurements from postmenopausal patients. We used ELISA to measure serum MDK levels and categorized patients into two groups based on T-scores (Fig. S1). Normal-BMD versus osteoporosis groups differed markedly in both hip and lumbar T-scores (Fig. 1B). There was no significant difference in age or body mass index between the two groups (Table S2). Serum MDK levels were significantly higher in the osteoporosis group compared with the non-osteoporosis group (Fig. 1C). Pearson correlation analysis revealed a negative correlation between serum MDK levels and hip and lumbar BMD T-scores (Fig. 1D and E).

We then generated an OVX mouse model and measured serum MDK levels in these mice using ELISA. The semi-quantitative analysis showed that bone microstructural parameters (BMD, Tb.Th, BV/TV, Tb.N, Tb.Sp, and BS) were significantly lower in the OVX group compared with the sham group (Fig. S2), confirming the successful establishment of the OVX model. Consistent with the human serum ELISA results, serum MDK levels in OVX mice were markedly higher than in the control group (Fig. 1F). Immunofluorescence analysis of bone sections revealed that MDK expression was predominantly localized in the bone marrow compartment. Quantitative assessment demonstrated significantly higher MDK signal intensity in control mice compared with OVX mice (Fig. 1G and H). This marrow-specific reduction of MDK expression in estrogen-deficient mice suggests a potential correlation between local MDK production and osteoporosis pathogenesis.

### iMDK alleviates bone loss via dual regulation of bone formation and inflammatory cytokines

**Figure 2:**
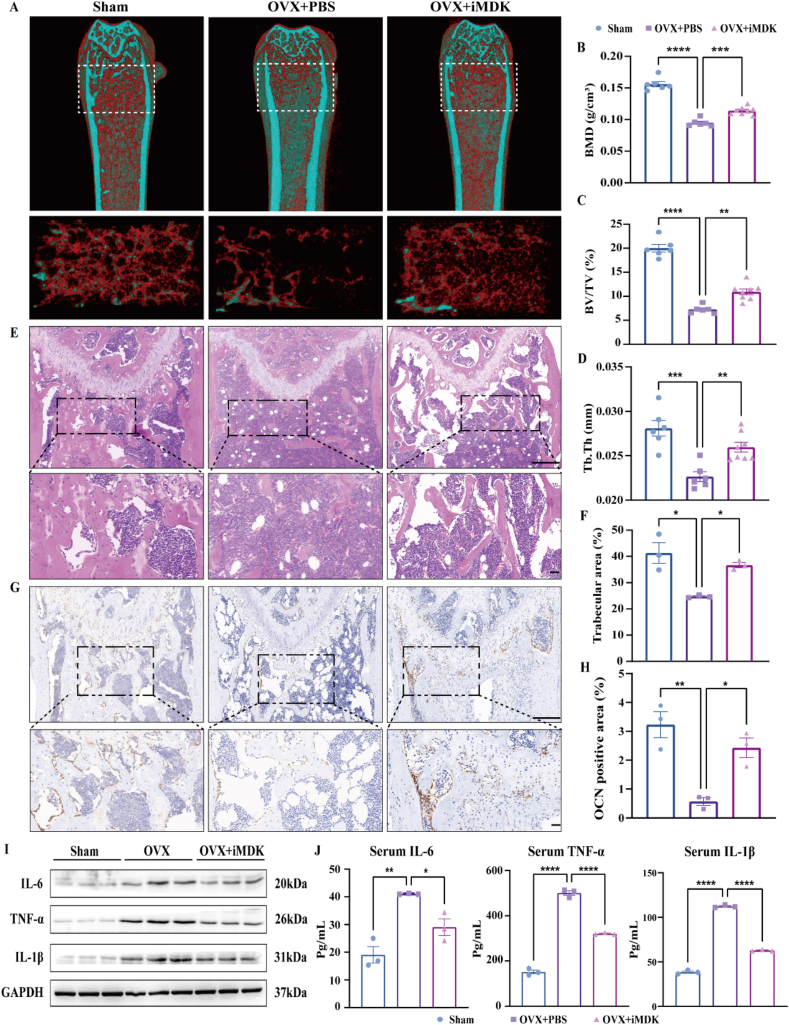
iMDK alleviates bone loss via dual regulation of bone formation and inflammatory cytokines. **(A)** Representative micro-CT images of distal femora (top) with three-dimensional reconstruction of the region of interest (bottom), with the blue indicating higher-density bone and the red indicating lower-density bone. **(B**–**D)** Statistical quantification of trabecular bone microstructural parameters (BMD, BV/TV, and Tb.Th). Inter-group comparisons were analyzed by one-way ANOVA. **(E)** Representative images of trabecular bone area in distal femur sections stained with hematoxylin and eosin. Scale bar, 200 μm or 50 μm. **(F)** Quantitative analysis of the trabecular bone area of the distal femur stained with hematoxylin and eosin. Inter-group comparisons were analyzed by one-way ANOVA. **(G)** Immunohistochemical staining of OCN in distal femurs. Scale bar, 200 μm or 50 μm. **(H)** Quantitative analysis of OCN-positive area. Inter-group comparisons were analyzed by one-way ANOVA. **(I)** Western blotting analysis of inflammatory cytokine expression (IL-6, TNF-α, and IL-1β) in mouse bone tissues. **(J)** Inflammatory cytokine expression (IL-6, TNF-α, and IL-1β) in mouse serum was detected by ELISA. Inter-group comparisons were analyzed by one-way ANOVA. ∗*p* < 0.05, ∗∗*p* < 0.01, ∗∗∗*p* < 0.001, and ∗∗∗∗*p* < 0.0001; “ns” indicates non-significant differences.

To better understand how MDK affects bone loss induced by estrogen deficiency, we established an OVX model and administered iMDK intraperitoneally. Figure S3 displays the molecular formula of iMDK, a small-molecule inhibitor targeting MDK. We administered intraperitoneal injections of either PBS or iMDK (9 mg/kg) three times a week for eight weeks following ovariectomy (Fig. S4). Micro-CT scans of the distal femur revealed that the OVX group exhibited a significant reduction in trabecular bone quantity and microstructural parameters (BMD, BV/TV, Tb.Th, Tb.N, and BS). Notably, the intraperitoneal injection of iMDK led to a dramatic increase in both trabecular bone quantity and microstructural parameters in the distal femur (Fig. 2A–D; Fig. S5). There was no significant difference in the microstructural parameters of cortical bone (Fig. S6).

We further assessed bone microstructure using histologic sections. Hematoxylin and eosin staining showed a significant reduction in the number of bone trabeculae in the OVX group, whereas the number of trabeculae in the iMDK group was significantly higher than in the OVX group (Fig. 2E and F). We performed immunohistochemical staining on bone tissue to detect OCN expression levels. OCN, a non-collagenous protein secreted by osteoblasts, serves as a marker of bone formation and plays a crucial role in bone mineralization and calcium binding. Zhang et al reported reduced OCN expression in the bone tissue of OVX mice, and our results are consistent with their findings.^30^ Our immunohistochemistry results showed that OCN expression in bone tissue was significantly higher in the iMDK treatment group compared with the OVX group (Fig. 2G and H).

Additionally, we observed that the inflammatory cytokines IL-6, IL-1β, and TNF-α were present in greater amounts in the bone tissue and serum of OVX mice. However, iMDK injection significantly reduced the levels of these cytokines (Fig. 2I and J; Fig. S7). These findings suggest that targeted inhibition of MDK significantly mitigates estrogen deficiency-induced bone loss by promoting bone formation and reducing inflammatory factor expression.

### Recombinant MDK protein dose-dependently inhibits osteogenic differentiation *in vitro*

**Figure 3:**
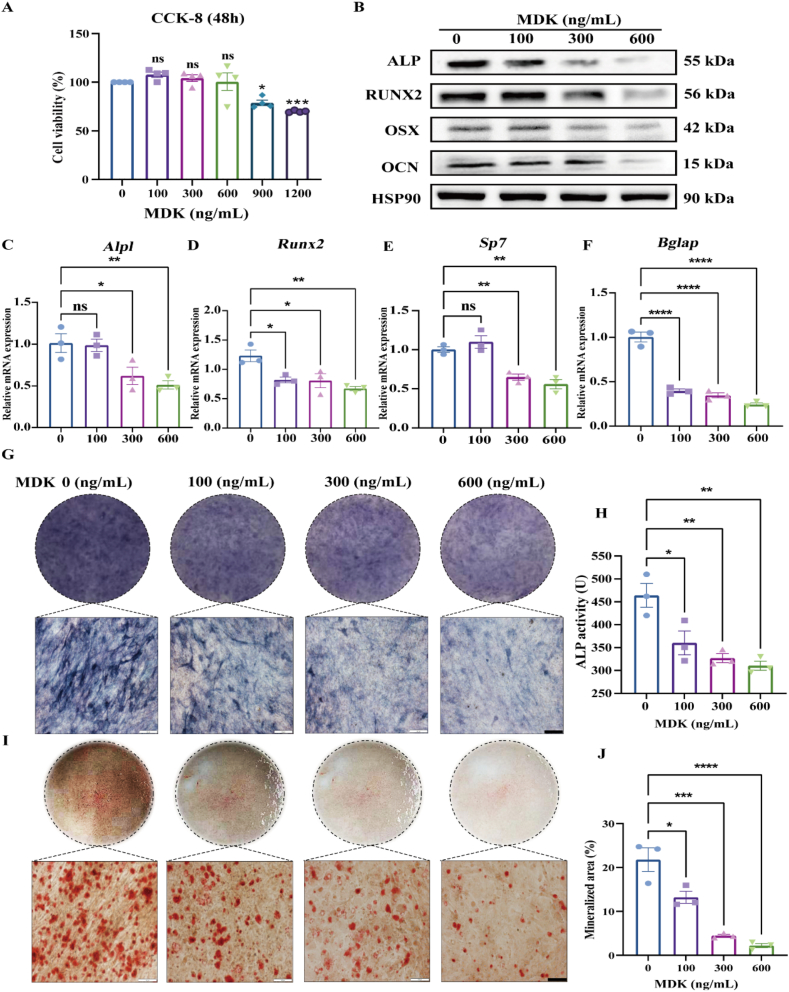
Recombinant MDK protein inhibits osteogenic differentiation *in vitro* in a dose-dependent manner. **(A)** Cell viability after treating MC3T3-E1 cells with recombinant MDK protein after 48 h, assessed using the CCK-8 assay. Inter-group comparisons were analyzed by one-way ANOVA. **(B)** Western blotting analysis of ALP, RUNX2, OSX, and OCN expression levels following MDK treatment (7 days). **(C–F)** Reverse transcription PCR analysis of mRNA expression levels of *Alpl*, *Runx2*, *Sp7*, and *Bglap* in MC3T3-E1 cells following MDK treatment (7 days). β-actin served as the internal control. Inter-group comparisons were analyzed by one-way ANOVA. **(G, H)** ALP staining and activity assays were performed after inducing MC3T3-E1 cells with recombinant MDK protein (0–600 ng/mL) for 14 days. Inter-group comparisons were analyzed by one-way ANOVA. **(I, J)** ARS staining and quantitative analysis were conducted after inducing MC3T3-E1 cells with recombinant MDK protein (0–600 ng/mL) for 21 days. Inter-group comparisons were analyzed by one-way ANOVA. Scale bar, 100 μm ∗*p* < 0.05, ∗∗*p* < 0.01, ∗∗∗*p* < 0.001, and ∗∗∗∗*p* < 0.0001; “ns” indicates non-significant differences.

We used the murine pre-osteoblastic cell line MC3T3-E1 to examine the toxicity of recombinant MDK protein. We treated MC3T3-E1 cells with various concentrations of MDK, ranging from 0 to 1200 ng/mL. The data indicated that MDK concentrations from 0 to 600 ng/mL did not affect cell viability. However, at concentrations of 900–1200 ng/mL, we observed significant inhibition of cell viability, suggesting potential cytotoxicity at higher doses (Fig. 3A). We then evaluated the effects of MDK at concentrations of 100–600 ng/mL on the expression of key osteogenic differentiation markers in MC3T3-E1 cells. Western blotting analysis showed that MDK treatment caused a dose-dependent down-regulation of several critical proteins involved in osteogenic differentiation, including ALP, runt-related transcription factor 2 (RUNX2), osterix (OSX), and OCN (Fig. 3; Fig. S8). Reverse transcription PCR analysis confirmed these findings, demonstrating a similar dose-dependent decrease in the mRNA levels of these osteogenic markers (Fig. 3C–F). To further validate the inhibitory effects of MDK on osteoblast differentiation, we conducted ALP staining and ARS staining to assess ALP activity and extracellular matrix mineralization, respectively. The results from these assays showed that MDK significantly suppressed both ALP activity (Fig. 3G and H) and matrix mineralization (Fig. 3I and J) in a dose-dependent manner in MC3T3-E1 cells. Collectively, these findings suggest that recombinant MDK protein inhibits osteoblast differentiation *in vitro* in a dose-dependent manner, likely through the down-regulation of key osteogenic differentiation markers.

### Transcriptome sequencing reveals a potential mechanism of recombinant MDK protein inhibiting osteoblast differentiation

**Figure 4:**
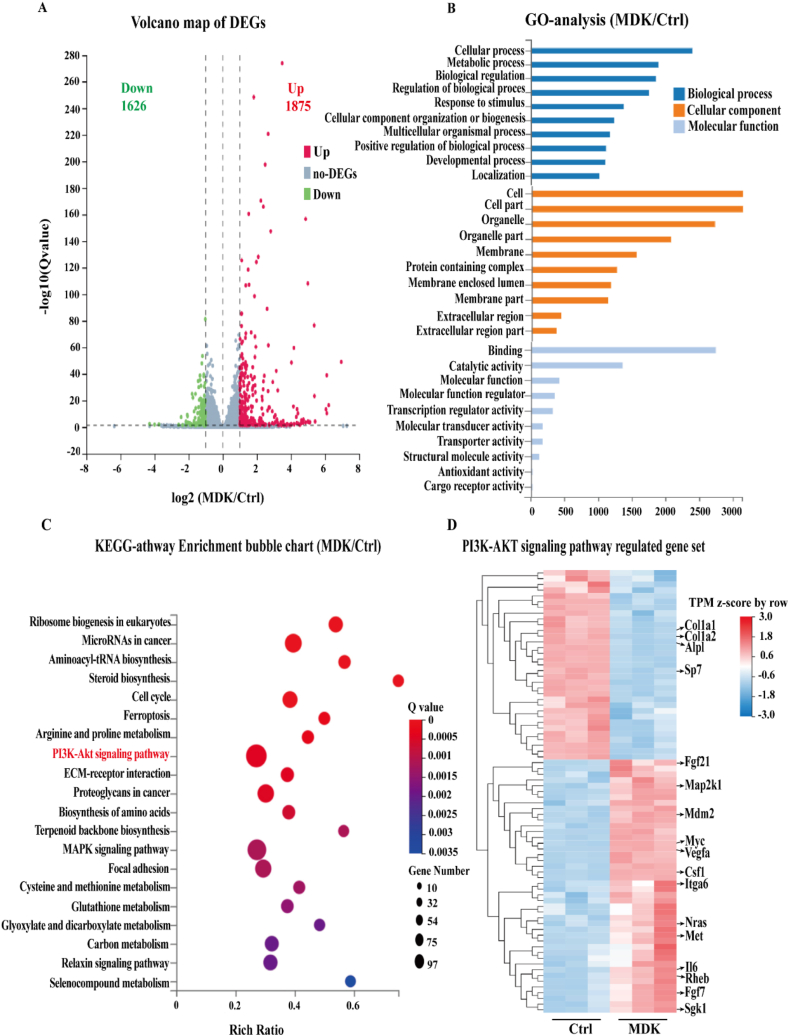
Transcriptome sequencing reveals the potential mechanism of recombinant MDK protein inhibiting osteoblast differentiation. **(A)** Volcano plot of differentially expressed genes (DEGs). The red dots represent significantly up-regulated genes (1875). The blue dots represent significantly down-regulated genes (1626). **(B)** Gene Ontology enrichment analysis of DEGs. **(C)** KEGG pathway enrichment bubble chart showing analysis of DEGs. **(D)** The representative heatmap showing hierarchical clustering analysis of representative DEGs. *p* < 0.05 indicates statistically significant differences.

To elucidate the inhibitory mechanism of MDK on osteogenic differentiation, we performed transcriptome sequencing on control MC3T3-E1 cells and those treated with recombinant MDK protein (600 ng/mL). Our analysis identified 3501 differentially expressed genes, with 1875 genes up-regulated, and 1626 genes down-regulated following MDK treatment. These results were revealed through differential gene expression and volcano plot analysis (Fig. 4A). Gene Ontology (GO) classification further categorized these differentially expressed genes into specific biological processes, cellular components, and molecular functions. The data showed significant enrichment in categories related to cellular processes, metabolic processes, and biological regulation. At the cellular level, most differentially expressed genes were associated with the cell, cell part, and organelle components, while binding and catalytic activity were the predominant molecular functions (Fig. 4B).

Additionally, Kyoto Encyclopedia of Genes and Genomes (KEGG) pathway enrichment analysis highlighted the PI3K/AKT signaling pathway as notably affected by MDK treatment (Fig. 4C). This pathway's activation was reflected in the up-regulation of several associated genes. Conversely, key osteogenic markers, such as *Alpl*, *Sp7*, and *Col1a1*, showed down-regulated expression in response to MDK treatment (Fig. 4D). These findings suggest that recombinant MDK protein may inhibit osteogenic differentiation by modulating the PI3K/AKT signaling pathway, leading to the down-regulation of essential genes involved in bone formation.

### MDK suppresses osteoblast differentiation via the PI3K/AKT signaling pathway

**Figure 5:**
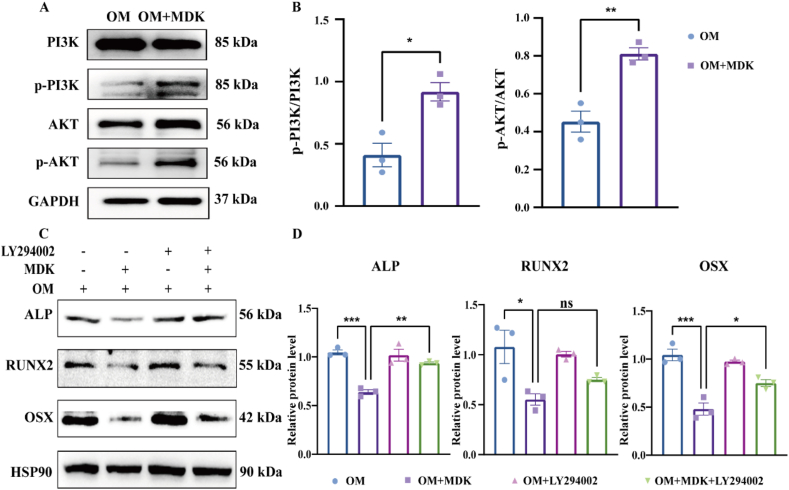
MDK suppresses osteoblast differentiation via the PI3K/AKT signaling pathway. **(A, B)** Western blot detection of the effect of recombinant MDK protein on the protein expression of molecules in the PI3K/AKT signaling pathway during the differentiation of MC3T3-E1 to osteoblasts (7 days). Inter-group comparisons were analyzed by a two-tailed unpaired Student's *t*-test (for normally distributed data with equal variance). **(C, D)** ALP, RUNX2, and OSX expression levels were detected by Western blotting. MC3T3-E1 cells were pretreated with 30 μM LY294002. Osteogenic differentiation was induced for 7 days. Inter-group comparisons were analyzed by one-way ANOVA. ∗*p* < 0.05, ∗∗*p* < 0.01, and ∗∗∗*p* < 0.001; “ns” indicates non-significant differences.

We conducted additional experiments to validate the phosphorylation status of key components within the PI3K/AKT signaling pathway. Western blotting analysis revealed that MC3T3-E1 cells treated with recombinant MDK protein showed significantly increased phosphorylation levels of both PI3K and AKT, as shown in Fig. 5A and B. To confirm these results, we used the PI3K inhibitor LY294002 in subsequent experiments. Western blotting analysis indicated that LY294002 treatment partially reversed the suppression of osteogenic-related protein expression induced by recombinant MDK protein. This reversal specifically affected proteins, such as ALP, RUNX2, and OSX, as illustrated in Fig. 5C and D. These findings strongly suggest that recombinant MDK protein inhibits osteoblast differentiation primarily through the activation of the PI3K/AKT signaling pathway. Notably, inhibiting this pathway with LY294002 partially counteracted the suppressive effect of recombinant MDK protein on osteoblast differentiation, underscoring the critical role of the PI3K/AKT pathway in this process.

### Recombinant MDK protein triggers the activation of inflammatory cytokines through the NF-κB signaling pathway

**Figure 6:**
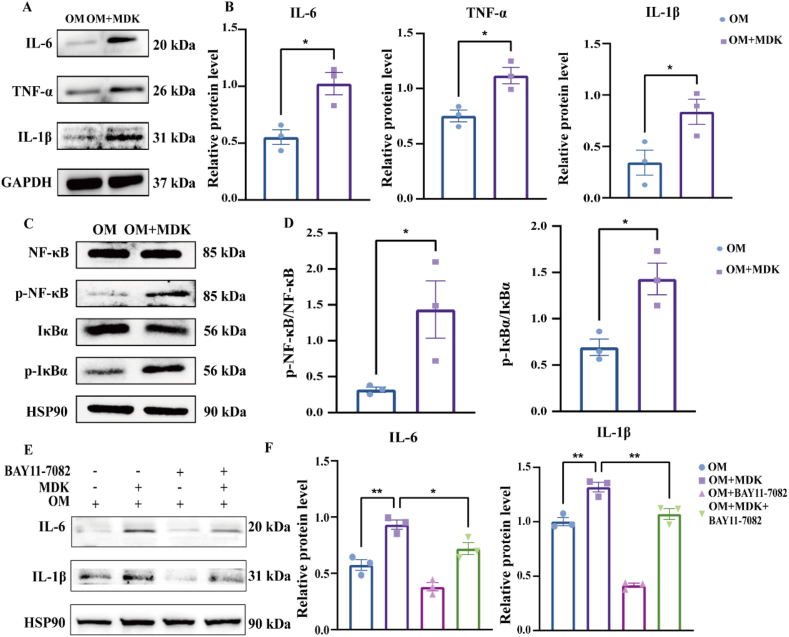
Recombinant MDK protein triggers the activation of inflammatory cytokines through the NF-κB signaling pathway. **(A, B)** IL-6, TNFα, and IL-1β expression levels were detected using Western blotting. MC3T3-E1 cells were treated with recombinant MDK protein (600 ng/mL). Osteogenic differentiation was induced for 7 days. Inter-group comparisons were analyzed by a two-tailed unpaired Student's *t*-test (for normally distributed data with equal variance). **(C, D)** Western blotting analysis of NF-κB signaling pathway molecules in MC3T3-E1 cells treated with recombinant MDK protein for 7 days during osteoblastic differentiation. Inter-group comparisons were analyzed by two-tailed unpaired Student's *t*-test (for normally distributed data with equal variance). **(E, F)** IL-6 and IL-1β expression levels were detected using Western blotting. MC3T3-E1 cells were pretreated with 10 μM BAY 11–7082. Osteogenic differentiation was induced for 7 days. Inter-group comparisons were analyzed by one-way ANOVA. ∗*p* < 0.05 and ∗∗*p* < 0.01.

**Figure 7:**
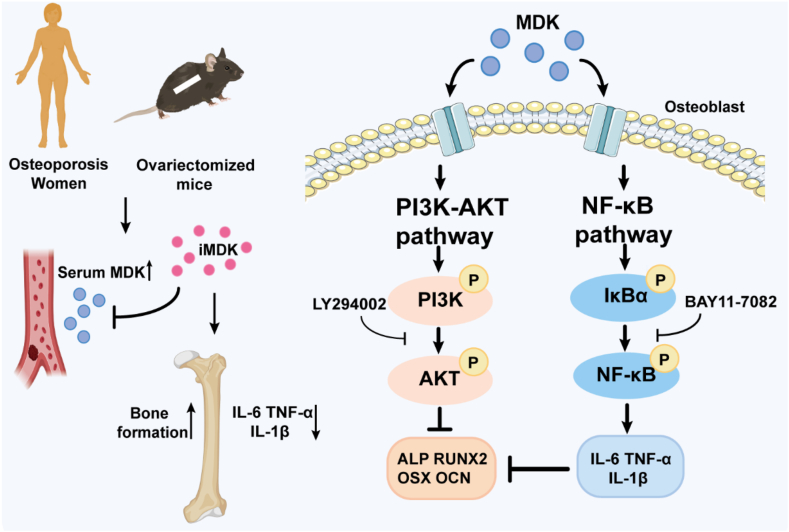
Schematic representation of MDK alleviating bone loss. MDK is significantly elevated in the serum of postmenopausal osteoporotic women and ovariectomized mice. Due to estrogen deficiency, iMDK alleviates bone loss by promoting bone formation and inhibiting inflammatory factors. Recombinant MDK protein inhibits osteogenic differentiation through the PI3K/AKT signaling pathway and up-regulates inflammatory factors IL-6, TNF-α, and IL-1β via the NF-κB signaling pathway.

Previous research has demonstrated that osteoblasts produce inflammatory cytokines, including TNF-α, IL-6, and IL-1β, in response to inflammatory stimuli, such as bacterial infection or mechanical stress.^31^, ^32^, ^33^, ^34^ We further investigated the regulation of inflammatory cytokines by MDK in osteoblasts. Treatment with recombinant MDK protein significantly increased the levels of TNF-α, IL-6, and IL-1β in MC3T3-E1 osteoblasts, as shown by Western blotting analysis (Fig. 6A and B). In addition, we observed the intracellular reactive oxygen species levels in MDK-treated MC3T3-E1 cells, and the results showed that MDK increased the intracellular reactive oxygen species levels in MC3T3-E1 cells (Fig. S9). Previous studies have indicated that recombinant MDK protein can activate the NF-κB signaling pathway,^23^^,^^35^^,^^36^ which is closely associated with inflammatory cytokines. Therefore, we assessed the phosphorylation levels of NF-κB p65. Western blotting analysis revealed a significant increase in the phosphorylation levels of NF-κB and Inhibitor of kappa B alpha (IκBα) in MC3T3-E1 cells treated with recombinant MDK protein (Fig. 6C and D). To determine if the NF-κB signaling pathway inhibitor BAY11-7082 could mitigate the MDK-induced up-regulation of inflammatory cytokines, we conducted additional experiments. Compared with the control group, BAY11-7082 partially reversed the up-regulation of IL-6 and IL-1β caused by recombinant MDK protein, as indicated by Western blotting analysis (Fig. 6E and F). These findings suggest that recombinant MDK protein activates the NF-κB signaling pathway, leading to an increase in the expression of inflammatory cytokines (see Fig. 7).

## Discussion

In recent years, the diagnosis, treatment, and prevention of osteoporosis have received substantial attention in clinical practice. As the population ages, osteoporosis prevalence rises, making it a central focus of medical research. This condition significantly affects patients' health and quality of life, underscoring the urgent need for enhanced diagnostic methods, effective treatments, and preventive strategies. In osteoporosis research, the exploration of growth factors and secretory proteins related to bone metabolism, their roles and mechanisms in the pathological process, and the development of targeted drugs have become primary research directions. Denosumab (an inhibitor of RANKL)^5^^,^^37^ and romosozumab (an inhibitor of SOST)^6^ have shown promising potential in osteoporosis research.

MDK is involved in cell growth, survival, angiogenesis, inflammation, and signal transduction. While MDK is expressed in multiple tissues, including the gastrointestinal tract, kidneys, spleen, lungs, and thyroid, its expression levels in normal tissues are generally weak compared with malignant tissues.^38^ MDK primarily functions in the nervous system, tumorigenesis, and inflammation. Previous studies have demonstrated its strong expression in the basal lamina of the cerebral cortex, where it exerts neuroprotective effects.^38^ Under pathological conditions, MDK is expressed in various tumor cell lines, functioning as both a cytokine and growth factor that promotes tumor cell proliferation, transformation, epithelial–mesenchymal transition, and angiogenesis.^39^ In adults, MDK is up-regulated in damaged tissues and participates in tissue repair processes. It also modulates inflammatory responses by facilitating leukocyte migration, inducing chemokine production, and suppressing regulatory T-cell activity.^40^ Notably, MDK expression has been detected in spindle-shaped mesenchymal stem cells at day 4 and chondrocytes within the chondrogenic ossification zone at day 7 post-fracture.^41^ As a growth factor and cytokine, MDK acts through binding to cell surface receptors. Haffner-Luntzer et al reported that MDK suppressed the expression of ALP, a key osteogenic differentiation marker, via interaction with the Wnt signaling receptor LRP-6.^42^ Liedert et al further revealed that MDK negatively regulated Wnt signaling in osteoblasts by binding to receptor-type protein tyrosine phosphatase zeta (Ptprz).^20^ Additionally, Muramatsu et al identified the α4β1 integrin complex as a functional receptor for MDK in osteoblasts.^43^ Early studies primarily explored MDK as a biomarker and therapeutic target for various cancers.^12^^,^^44^ Recently, research has increasingly focused on MDK's role in chondrogenesis, skeletal development, and bone remodeling.^19^^,^^20^^,^^45^ Our study reveals a significant inverse association between serum MDK levels and BMD, suggesting that MDK may contribute to the development of postmenopausal osteoporosis. Furthermore, we found that serum MDK levels were significantly higher in individuals with osteoporosis compared with those without the disease. Subsequently, we established an OVX mouse model and observed that serum MDK levels in OVX mice were significantly higher than those in controls, while immunofluorescence results of bone sections revealed that MDK expression in the bone marrow of OVX mice was lower than that of controls. We hypothesized that this difference might be the result of a combination of changes in the local metabolic environment of bone tissue as well as systemic responses. Ovariectomy-induced osteoporosis is usually accompanied by a systemic stress and inflammatory response,^3^ which may prompt other tissues or organs (*e.g.*, the liver or the immune system) to secrete more MDK proteins into the peripheral blood, resulting in higher serum concentrations. Based on these findings, we investigated whether lowering MDK levels could potentially mitigate bone loss caused by ovariectomy.

iMDK, a small molecule inhibitor that targets MDK and inhibits the PI3K-AKT and MAPK pathways, suppresses endogenous MDK expression and has shown therapeutic efficacy in tumor trials. However, its use in treating osteoporosis has not yet been documented.^14^, ^15^, ^16^, ^17^, ^18^ In our animal experiments, we found that iMDK increased OCN expression in the bone tissues of OVX mice and partially reversed the decrease in BMD and trabecular bone loss in these mice. Therefore, iMDK may represent a promising new treatment for postmenopausal osteoporosis. Our immunohistochemical staining results showed a decrease in OCN expression in the bone tissue of OVX animals compared with the control group. This result aligns with the findings of Zheng Zhang et al,^30^ but contrasts with those of Yan Zhang et al.^46^ We hypothesize that this discrepancy may stem from various factors, including differences in experimental procedures, post-modeling intervention strategies, and mouse strains used. Additionally, subtle variations in experimental conditions, such as drug administration timing, dosage, and detection methods, might also contribute to the differing results. In our *in vitro* experiments, treating osteoblast precursor cells MC3T3-E1 with recombinant MDK protein inhibited osteogenic differentiation, ALP activity, and osteoblast matrix mineralization. These results suggest that recombinant MDK protein negatively regulates bone formation by inhibiting osteoblast activity.

The PI3K/AKT pathway is crucial for regulating the differentiation of osteoblasts and osteoclasts, maintaining the balance of bone metabolism, and significantly influencing the development and progression of osteoporosis.^47^ Additionally, triptolide inhibits the PI3K/AKT signaling pathway to prevent glucocorticoid-induced osteoporosis.^47^ MiR-483-5p suppresses SATB2 and activates the PI3K/AKT pathway,^48^ both of which contribute to the pathophysiology of postmenopausal osteoporosis. Vitexin regulates angiogenesis and osteogenesis in ovariectomized rats via the VDR/PI3K/AKT/eNOS signaling pathway.^49^ Furthermore, studies have highlighted a close relationship between MDK and the PI3K/AKT pathway.^22^^,^^24^ Our RNA sequencing results showed that the PI3K/AKT pathway was significantly enriched in the genes with altered expression. In *in vitro* experiments, recombinant MDK protein increased the phosphorylation levels of PI3K and AKT in MC3T3-E1 cells. Moreover, the PI3K/AKT inhibitor LY294002 was able to counteract the inhibition of osteogenic differentiation-related proteins caused by MDK, suggesting that recombinant MDK protein activates the PI3K/AKT signaling pathway and impedes osteoblast development.

Inflammation, a key factor underlying osteoporosis, promotes bone resorption^7^^,^^50^ and inhibits bone formation,^4^^,^^51^^,^^52^ leading to disruptions in bone metabolism and ultimately resulting in osteoporosis. Inflammatory cytokines, such as TNF-α, IL-1, and IL-6, regulate the balance between bone production and bone resorption.^53^^,^^54^ Osteoblasts release these inflammatory cytokines, including TNF-α, IL-1β, and IL-6, in response to bacterial infection, mechanical stress, or other pro-inflammatory stimuli. These cytokines enhance osteoclast activity and directly or indirectly affect osteoblast development.^31^, ^32^, ^33^, ^34^ Stashenko et al reported that IL-1β could inhibit osteoblast function and reduce the expression of collagen and alkaline phosphatase.^55^ Gilbert et al found that TNF-α suppressed RUNX2, a crucial regulator of osteoblast development, by affecting mRNA stability and transcription inhibition.^56^ Additionally, IL-6 can inhibit Wnt signaling in osteoblasts and synovial fibroblasts.^57^

Certain theories suggest that MDK plays a significant role in inflammation and is associated with inflammatory diseases such as Crohn's disease, pathological angiogenesis, and autoimmune conditions like experimental autoimmune encephalomyelitis.^58^, ^59^, ^60^ A deficiency in MDK can decrease mice's susceptibility to osteoarthritis. Maruyama et al have reported that MDK is crucial for the development of rheumatoid arthritis by promoting the migration of inflammatory leukocytes.^25^ Our findings indicate that serum and bone tissues from OVX mice exhibit elevated levels of inflammatory cytokines (IL-6, TNF-α, and IL-1β); however, treatment with iMDK resulted in a significant reduction in their levels. In our *in vitro* study, recombinant MDK protein increased the production of inflammatory cytokines (IL-6, TNF-α, and IL-1β) in MC3T3-E1 cells. These results suggest that recombinant MDK protein stimulates the release of inflammatory cytokines in osteoblast precursor cells.

The NF-κB signaling pathway is a key regulator of the inflammatory response. In its inactive state, the NF-κB complex is bound to IκB proteins, which sequester it in the cytoplasm. Upon activation, the NF-κB complex dissociates from IκB and translocates to the nucleus, where it regulates the expression of genes that produce inflammatory cytokines, thereby controlling the inflammatory response.^61^ Relevant studies have shown that MDK can directly activate the NF-κB signaling pathway in NSCLC and breast cancer cells.^23^^,^^36^ However, there is limited information on whether MDK directly activates the NF-κB signaling pathway in osteoblasts. To address this gap, we examined the phosphorylation statuses of proteins involved in the NF-κB signaling cascade. Our findings revealed that recombinant MDK protein increased the phosphorylation levels of NF-κB and IκBα. Furthermore, the NF-κB pathway inhibitor BAY11-7082 partially reduced the increase in inflammatory cytokines induced by recombinant MDK protein, indicating that recombinant MDK protein enhances the production of inflammatory cytokines by activating the NF-κB signaling pathway.

In conclusion, our study established a significant correlation between serum MDK levels and osteoporosis in postmenopausal women. We found that the small-molecule MDK inhibitor, iMDK, effectively mitigated estrogen deficiency-induced bone loss through dual actions—promoting bone formation and inhibiting inflammatory cytokines. Our *in vitro* experiments demonstrate that MDK suppresses osteoblast differentiation by activating the PI3K-AKT pathway and increases inflammatory cytokine expression via activation of the NF-κB signaling pathway. This research offers valuable insights for identifying potential therapeutic targets and developing new treatment strategies for osteoporosis.

## CRediT authorship contribution statement

**Xieyidai Ruze:** Writing – review & editing, Writing – original draft, Project administration, Methodology, Investigation, Conceptualization. **Yutong Hu:** Methodology, Conceptualization. **Xiongyi Wang:** Resources. **Houfu Lai:** Funding acquisition, Formal analysis. **Ruizhi Zhang:** Supervision, Software, Resources, Conceptualization. **Sheng Pan:** Methodology, Investigation. **Jiajun Zhang:** Validation, Supervision, Conceptualization. **Yike Wang:** Formal analysis. **Simin Yun:** Conceptualization. **Ying Xu:** Visualization, Methodology, Investigation. **Junjie Li:** Writing – review & editing, Methodology, Investigation, Conceptualization. **Youjia Xu:** Supervision, Funding acquisition.

## Funding

This work was supported by the Natural Science Foundation of China (No. 82372455), Jiangsu Provincial Medical Key Laboratory Cultivation Unit (China) (No. JSDW202254), Special Project of "Technological Innovation" Project of CNNC Medical Industry Co., Ltd., China (No. ZHYLZD2023001), and Zhejiang Provincial Natural Science Foundation of China (No. LBZ24H060001).

## Conflict of interests

All authors declared no conflict of interests.
