# Supplementary material for: Targeting MDK alleviates bone loss via dual regulation of osteogenic differentiation and inflammatory cytokine expression
Source: Genes Dis. 2025 Nov 10;13(3):101931. doi: 10.1016/j.gendis.2025.101931 (PMC12886531; doi:10.1016/j.gendis.2025.101931)
Supplement: Multimedia component 1 [file mmc1.docx]

Supplementary Materials for

**Targeting MDK alleviates bone loss *via* dual regulation of osteogenic differentiation and inflammatory cytokines expression**

*Xieyidai Ruze^1#^, Yutong Hu^1#^, Xiongyi Wang^1#^, Houfu Lai^1^, Ruizhi Zhang^1^, Sheng Pan^1^, Jiajun Zhang^1^, Yike Wang^1^, Simin Yun^1^, Ying Xu^3***^, Junjie Li^1,2**^, Youjia Xu^1*^*

* **Corresponding author**: Department of Orthopedics, Department of Osteoporosis, The Second Affiliated Hospital of Soochow University, No.1055 Sanxiang Road, Suzhou, 215000, China.

** **Corresponding author**: Department of Orthopedic Surgery, The First Affiliated Hospital of Zhejiang Chinese Medical University (Zhejiang Provincial Hospital of Chinese Medical), Hangzhou, 310000, China.

*** **Corresponding author**: Jiangsu Key Laboratory of Neuropsychiatric Diseases and Cambridge-Su Genomic Resource Center, Medical College of Soochow University, Suzhou, 215000, China

**This PDF file includes:**

Figs. S1 to S9

Tables. S1-S2


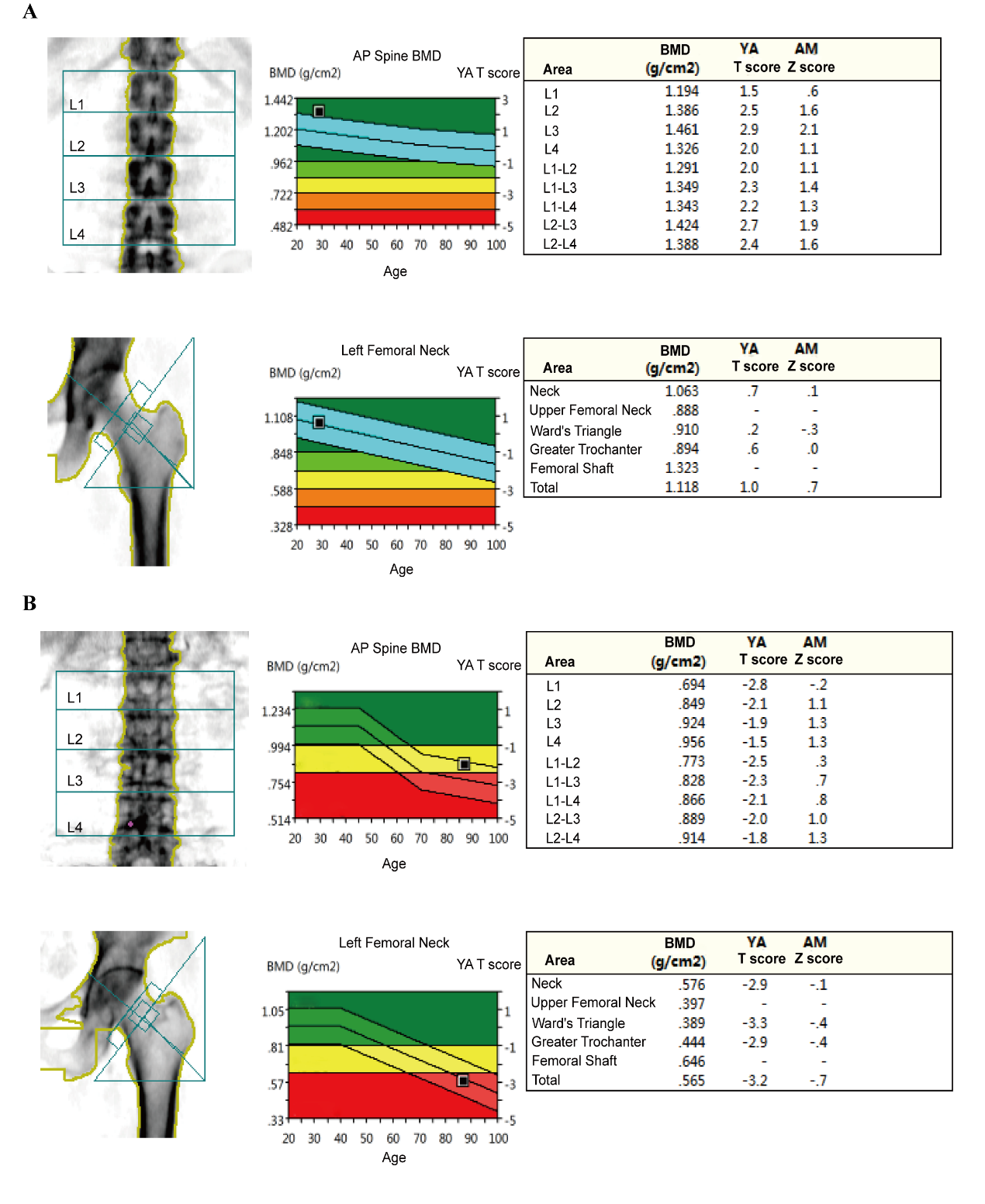


**Fig. S1.** Representative DXA reports from the non-osteoporotic group (A) and the osteoporotic group (B).


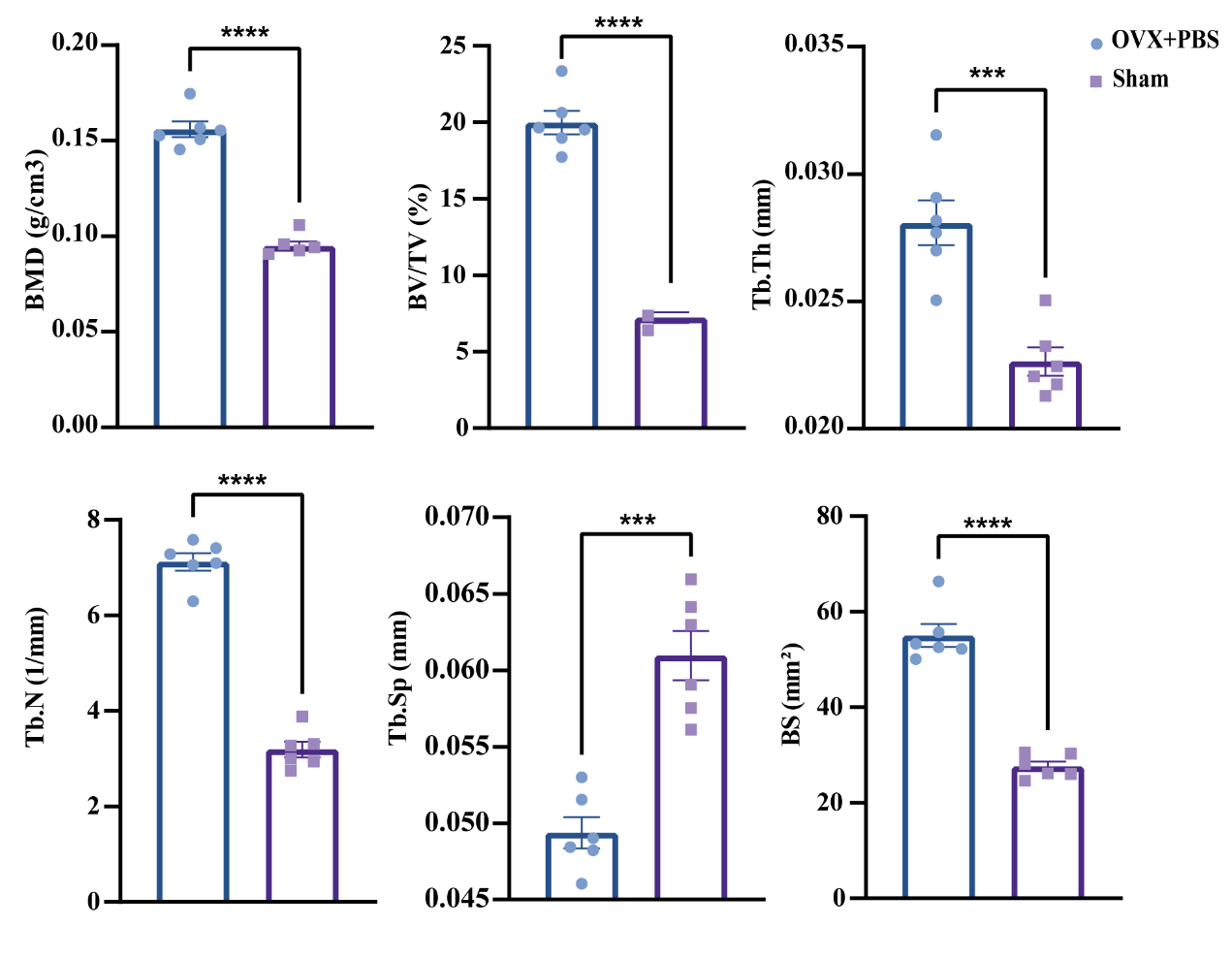


**Fig. S2.** Statistical quantification of trabecular bone microstructural parameters in OVX mice (BMD, BV/TV, Tb.Th, Tb.N, Tb.Sp, and BS), Inter-group comparisons were analyzed by two-tailed unpaired Student's t-test (for normally distributed data with equal variance). ****P* < 0.001, *****P* < 0.0001.


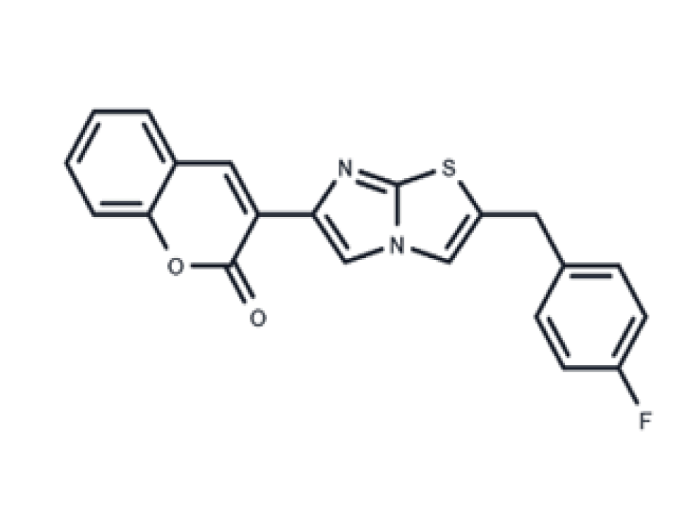


**Fig. S3.** Molecular formula of iMDK.


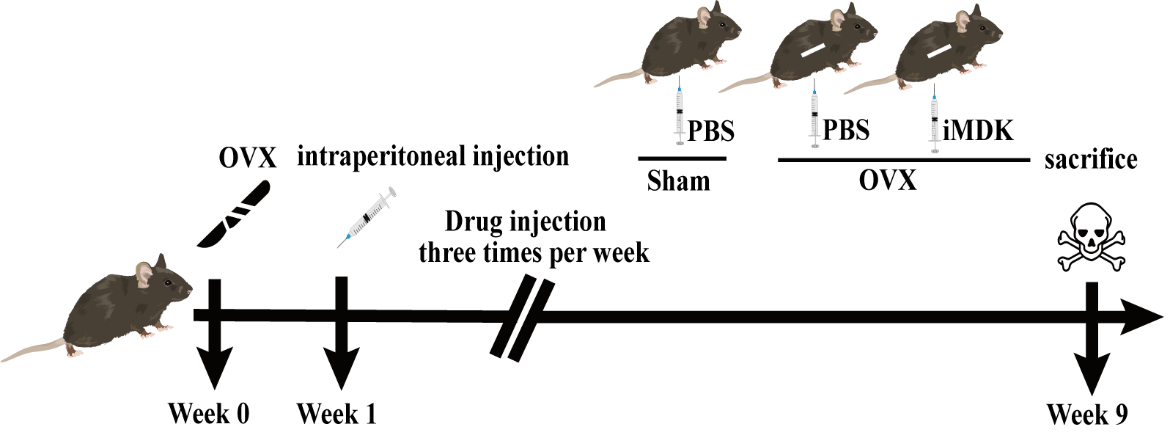


**Fig. S4.** Workflow diagram of the animal experiment.


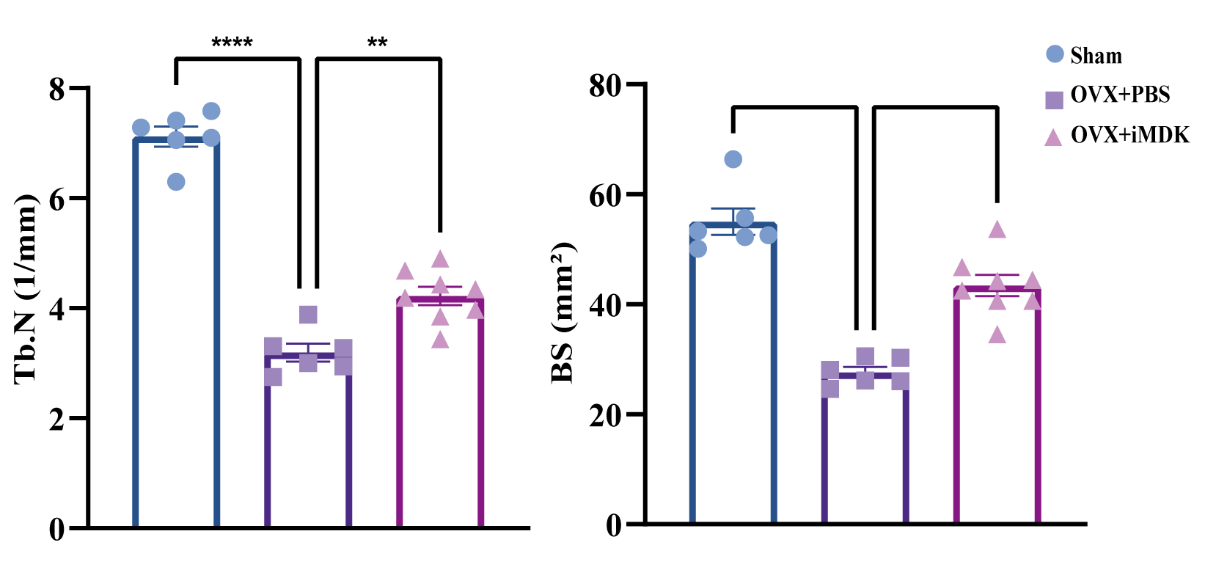


**Fig. S5.** Statistical quantification of trabecular bone microstructural parameters (Tb.N and BS), Inter-group comparisons were analyzed by one-way ANOVA. ***P* < 0.01, *****P* < 0.0001, ns indicates non-significant differences.


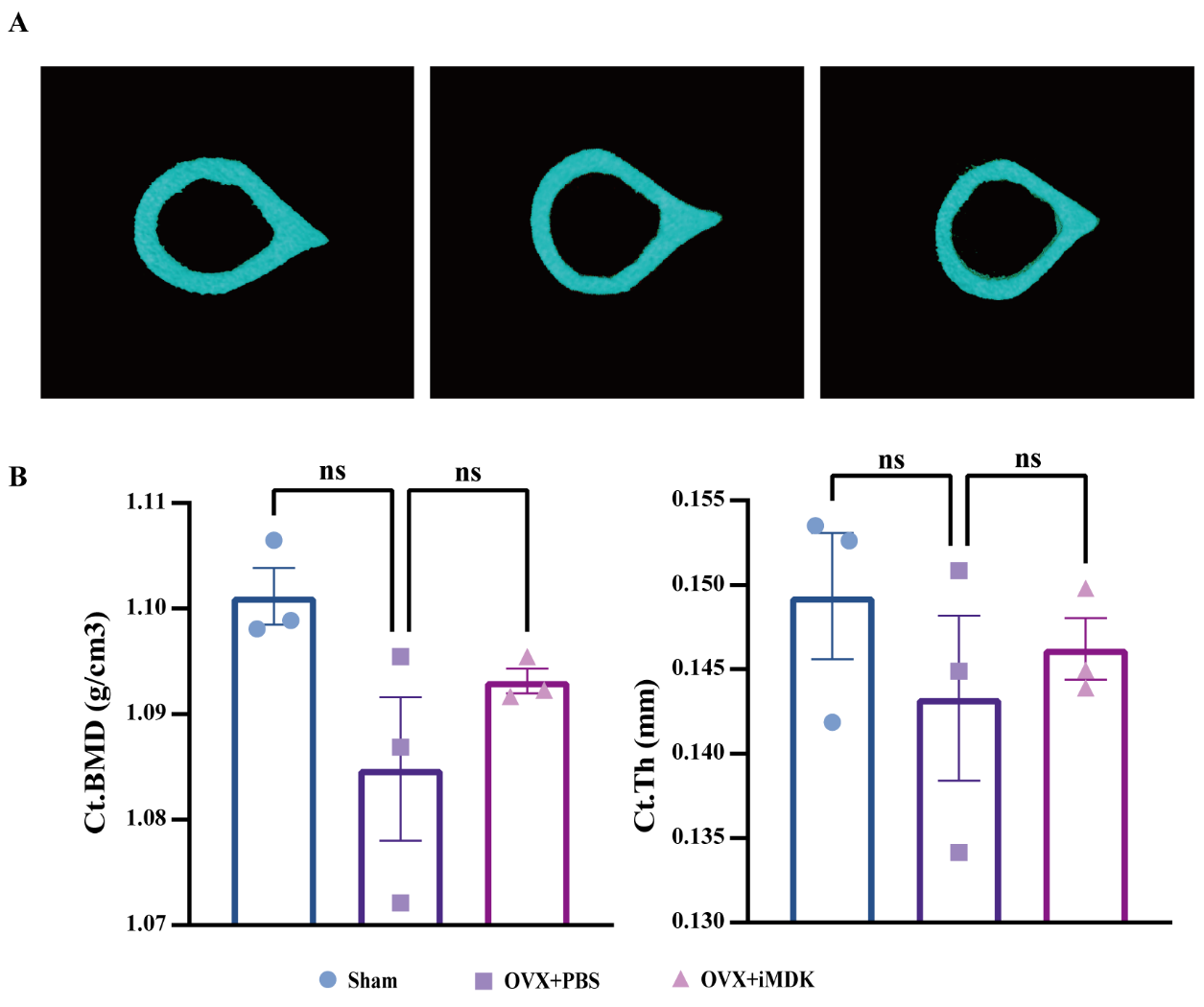


**Fig. S6.** A. Representative 3D reconstruction images from micro-CT scans of the distal femur cortical bone. B. Statistical quantification of cortical bone microstructural parameters (Ct.BMD and Ct.Th), Inter-group comparisons were analyzed by one-way ANOVA. ns indicates non-significant differences.


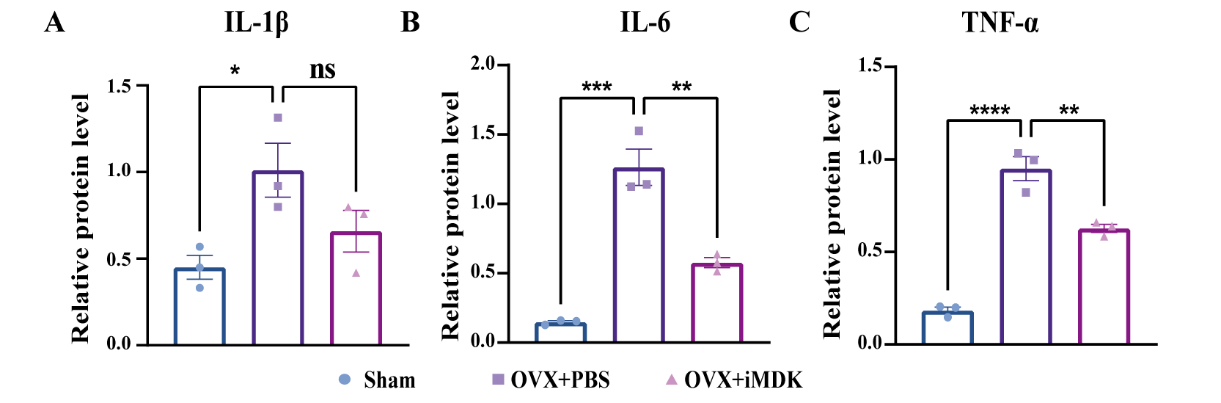


**Fig. S7.** Statistical quantification of expression levels of inflammatory cytokines in bone tissue assessed using Western blot, Inter-group comparisons were analyzed by one-way ANOVA. **P* < 0.05, ***P* < 0.01, ****P* < 0.001, ns indicates non-significant differences.


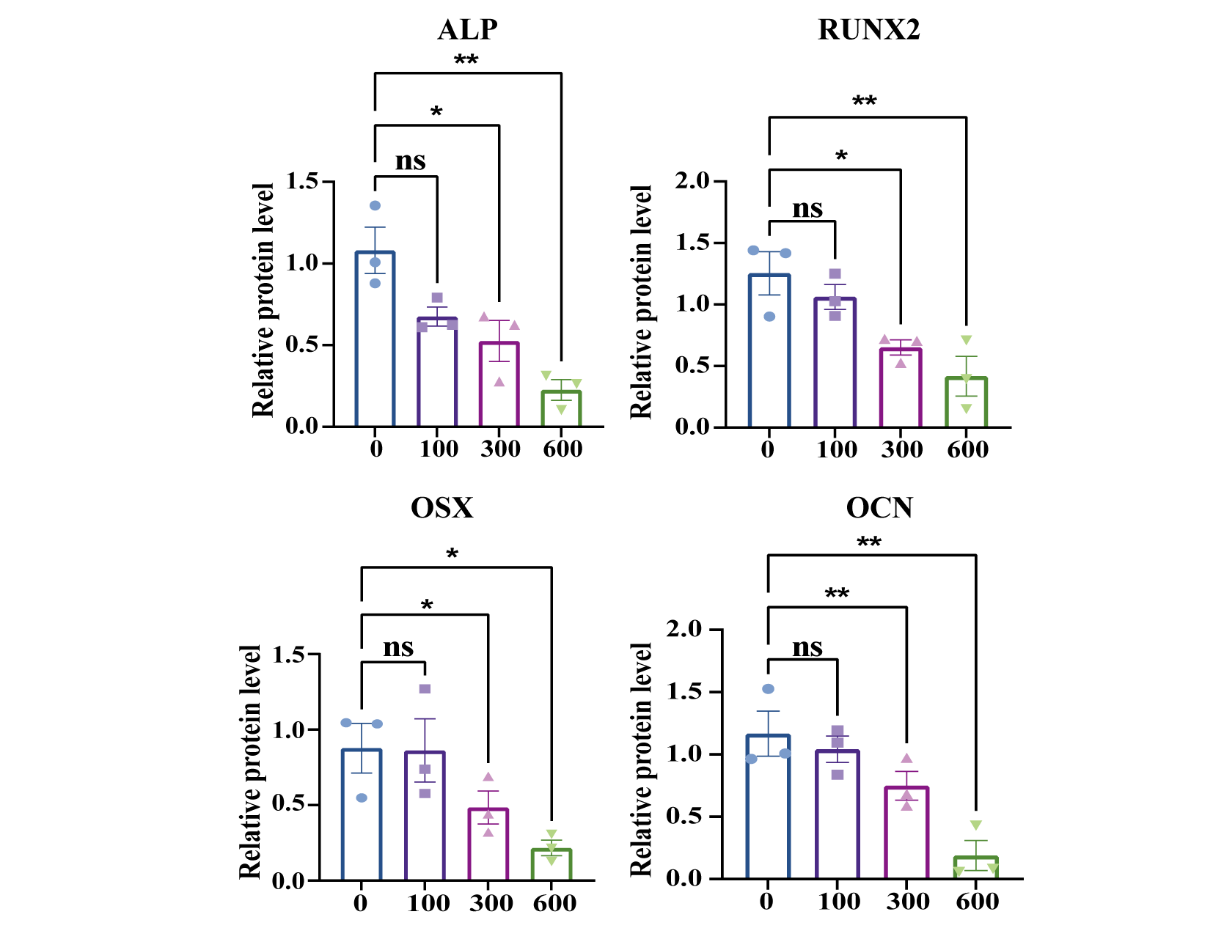


**Fig. S8.** Statistical quantification of expression levels of ALP, RUNX2, OSX, and OCN assessed using Western blot, Inter-group comparisons were analyzed by one-way ANOVA. **P* < 0.05, ***P* < 0.01, ns indicates non-significant differences.


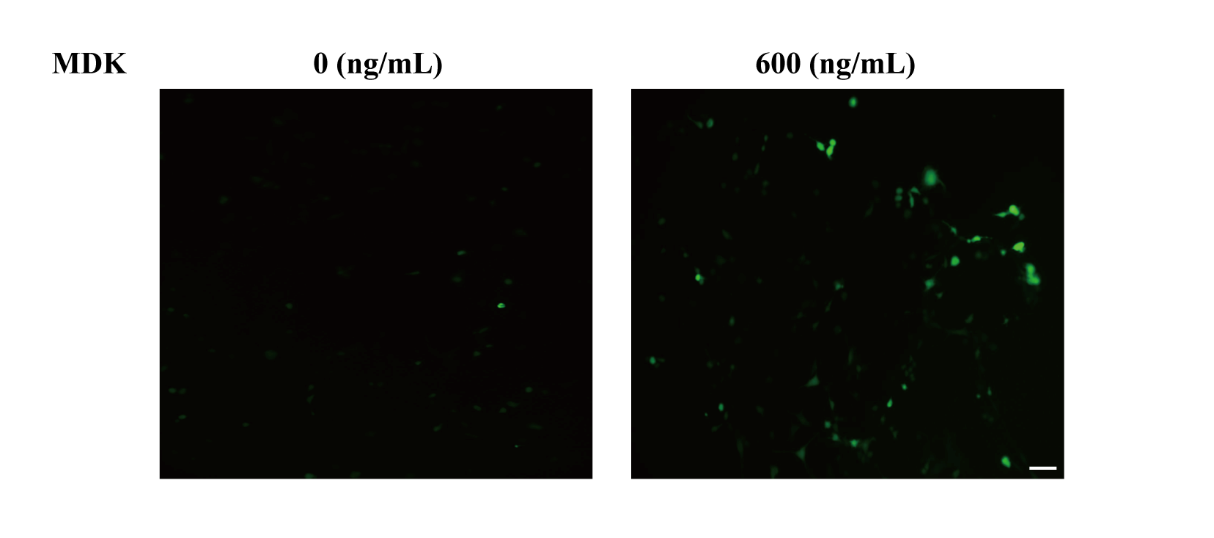


**Fig. S9.** Intracellular reactive oxygen species were observed after treating cells with 600 ng/mL of recombinant MDK protein for 2h. Scale bar, 200 μm.

**Table S1.** Comparison of baseline data between the non-osteoporosis group and the osteoporosis group ($\bar{X}\pm S$)

|  | Number | Age (years) | BMI (kg/m²) | T-scores | |
| --- | --- | --- | --- | --- | --- |
|  |  |  |  | Hip | Lumbar |
| Non-OP | 42 | 64.48 ± 6.23 | 26.30 ± 2.22 | 0.14 ± 0.62 | 0.77 ± 1.01 |
| OP | 42 | 66.90 ± 7.80 | 25.44 ± 1.73 | -2.59 ± 0.63 | -3.05 ± 0.87 |
| *t* | - | 1.557 | 1.958 | 19.72 | 18.39 |
| *P* | - | 0.1233 | 0.0536 | < 0.001 | < 0.001 |

Non-OP: non-osteoporosis group, OP: osteoporosis group, BMI: body mass index. *P* < 0.05 indicates statistically significant differences.

**Table S2.** RT-qPCR primer sequences

| Gene | Primer Sequence (5’-3’) |
| --- | --- |
| *Gapdh*-F  *Gapdh-*R | ACTGAGGACCAGGTTGTC  TGCTGTAGCCGTATTCATTG |
| *Alp*-F  *Alp*-R | AACCCAGACACAAGCATTC  AACCCAGACACAAGCATTCG |
| *Runx2*-F  *Runx2*-R | GGTACTTCGTCAGCATCCTATCAG  GCTTCCGTCAGCGTCAACAC |
| *Sp7*-F  *Sp7*-R | ATGGCGTCCTCTCTGCTTG  TGAAAGGTCAGCGTATGGCTT |
| *Bglap*-F  *Bglap*-R | GAACAGACAAGTCCCACACAGC  TCAGCAGAGTGAGCAGAAAGAT |
